# Intra-arterial catheter-directed CT angiography for assessment of endovascular aortic aneurysm repair

**DOI:** 10.1371/journal.pone.0221375

**Published:** 2019-09-10

**Authors:** Marco V. Usai, Mirjam Gerwing, Antje Gottschalk, Peter Sporns, Walter Heindel, Alexander Oberhuber, Moritz Wildgruber, Michael Köhler

**Affiliations:** 1 Department of Vascular and Endovascular Surgery, University Hospital Münster, Münster, Germany; 2 Department of Clinical Radiology, University Hospital Münster, Münster, Germany; 3 Department of Anesthesiology, Intensive Care and Pain Medicine, Münster, University Hospital Münster, Germany; Medical University of Vienna, AUSTRIA

## Abstract

**Objective:**

To compare the efficacy and safety as well as associated image quality of catheter-directed CT angiography (CCTA) with a low dose of iodine contrast agent compared to intravenous CTA in patients undergoing endovascular aortic aneurysm repair (EVAR).

**Methods:**

Retrospective data analysis of 92 patients undergoing EVAR between January 2009 and December 2017 was performed. Patients were divided in two groups; those receiving CTA (n = 59) after intravenous contrast agent application and those receiving CCTA (n = 33) via an intraarterial catheter placed in the descending aorta. Demographic and cardiovascular risk factors as well as renal function parameters before, immediately after and 6–60 months after EVAR were evaluated. As primary endpoint, changes in serum creatinine levels in the two groups were evaluated. Secondary endpoints encompassed complications associated with intraarterial catheter placement. Objective (signal-to-noise ratios) and subjective image quality (5-point Likert scale) were compared.

**Results:**

Amount of contrast medium was significantly lower in CCTA compared to i.v. CTA (23 ± 7 ml vs. 119 ± 15 ml, p<0.0001). Patients undergoing catheter-directed CTA had higher baseline creatinine values compared to the group with intravenous iodine application (1.9 ± 0.6 mg/dl vs. 1.3 ± 0.5 mg/dl; p<0.0001). Follow-up serum creatinine levels however did not show significant alterations between the two groups (1.9 ± 0.4 mg/dl vs. 1.3 ± 0.5 mg/dl). No major complications were detected in the CCTA group. Signal-to-noise ratio (SNR) was comparable between i.v. CTA and CCTA (8.5 ± 4.6 vs. 7.7 ± 4.0; p = 0.37) and subjective image similarly revealed no differences with a good interobserver agreement (ICC = 0.647).

**Conclusions:**

Catheter-directed CTA is safe and provides comparable image quality with a substantial retrenchment of the needed amount of iodine-based contrast medium. However, no benefit of the reduced contrast medium protocol with respect to renal function was observed.

## Introduction

Abdominal aortic aneurysm is a common pathologic finding especially in males over the age of 65 with a prevalence of 4–7% [1, 2]. If ruptured, mortality rates are high, between 65–85% [3]. Therefore, the guidelines suggest treatment if the maximum diameter exceeds 55 mm or a rapid growth of the aneurysm is noted [4] While open repair can be offered to patients who are otherwise healthy, the endovascular approach is a less invasive procedure, especially of benefit for patients with severe comorbidities [5]. In a meta-analysis of four trials, including a total of 2783 patients, an early survival advantage in the EVAR group compared to the open repair group was found for the first three years, followed by a period of a significantly higher aneurysm-related mortality in the EVAR group [6].

A detailed preoperative planning and assessment of the aortic anatomy, including the correct measurement of diameters, is mandatory to reduce the risk of procedure related complications. This is, in most cases, achieved with an intravenous computed tomography angiography (CTA).

Overall, renal complications occur in 1% of patients after EVAR and 5% of patients after open elective repair of an infrarenal abdominal aortic aneurysm. Although it is thus a fairly rare complication, it results in an increased 30-day mortality. Predictors of renal complications include elevated baseline GFR, open approach, transfusion and prolonged operative time [7].

This problem is especially severe in octogenarians, associated with high failure to rescue rates. Thus, Dang et al. established an interactive risk calculator of acute kidney injury (AKI) after abdominal aortic aneurysm repair, including the independent risk factors chronic obstructive pulmonary disease, chronic kidney disease stage III, peripheral artery disease, preoperative β –blocker use and aneurysm diameter [8]. In the long-term follow up, AKI is less likely to occur after EVAR, while these patients experience a significant but delayed decline in GFR over time compared to open repair [9].

Following the European Society for Vascular Surgery clinical practice guidelines on the care of patients with an abdominal aortic aneurysm, intravenous CTA is conducted preoperatively to plan the intervention, and postoperatively to ensure the treatments’ success and later to follow-up and identify possible endoleaks [10]. If manageable, patients with impaired renal function should thus only be exposed to as little iodinated contrast agent as possible [11]. For this reason, a low-dose contrast medium protocol has been proposed for patients with impaired renal function by receiving an intra-aortic catheter for CTA [12–14].

In this retrospective study we aimed to compare safety and efficacy as well as associated image quality of CCTA compared to standard i.v. CTA in patients undergoing EVAR for the treatment of aortic aneurysms. We aimed to investigate if the reduced amount of contrast media applied via the intraarterial route has an actual benefit for the kidney function in the course after EVAR procedures and if this benefit really outweighs the associated risks caused by the intraarterial catheter placement.

## Materials and methods

### Study characteristics

This is a retrospective single center study at a tertiary care university hospital, of 92 patients undergoing EVAR between January 2009 and December 2017. The study was approved by the local ethics committee of the Westfälische Wilhelms Universität Münster (protocol number 2017-464-f-S). Due to the retrospective character of the study informed consent was waived by the institutional review board. Two consecutive groups of patients were analyzed; those receiving an intravenous CTA (n = 59) between January 2013 and December 2014 and those receiving a catheter-directed CTA (n = 33) between January 2009 and December 2017. Indication for catheter-directed CTA was a creatinine level > 1.4mg/dl and at least one additional risk factor or sign of acute/chronic kidney injury. Patients with terminal CKD and a residual excretion of less than 100 ml/d, already on dialysis, were excluded. Demographic and cardiovascular risk factors were collected, nephrotoxic drugs intake including angiotensin I and II blocker, diuretics and anti-inflammatory medication. Moreover, SCr values before the preoperative CTA, after CTA, before surgery and late at range 6–60 months after EVAR were analyzed. The contrast agent volume for every CTA, routinely performed before, immediately after and during follow-up, was documented.

### Intravenous CTA

After a survey scan, a region of interest (ROI) was placed in the aorta for bolus tracking, which automatically starts the CT scan when a threshold of 100 HU is reached. Depending on the assessed body part (thoracoabdominal aorta or only abdominal aorta), the ROI was placed either in the aortic arch or in the suprarenal visceral segment, respectively. Application of contrast medium (100–150 ml, depending on the weight and constitution of the individual patient) was done via a peripheral venous catheter connected to a standard injector. An injection rate of 5 ml/s with a delay of 10s was chosen. Following contrast media injection, a bolus of 50 ml sodium chloride was applied. Preoperatively an arterial CT scan only was conducted. For follow up after EVAR, a native scan over the stentgraft was followed by an arterial and venous scan, to detect possible endoleaks.

### Catheter-directed CTA

Brachial access was chosen instead of femoral access due to immediate mobilization of the patient and no need for bed rest after groin puncture. For brachial catheter placement the puncture site was infiltrated with 2% xylocaine, an 18-G needle was used for vessel puncture. Ultrasound-guided puncture was performed when necessary. After the insertion of a 4-F arterial sheath in Seldinger technique a pigtail catheter was navigated into the aorta with the help of a 0.035 guidewire striving to avoid contact with the ostia of the cerebrovascular vessels. The catheter was either placed in the aortic arch for the assessment of thoracoabdominal aneurysms or the suprarenal visceral segment for abdominal aneurysms. Correct intraarterial placement was verified by aspirating arterial blood, contrast injections were avoided during catheter placement. For the CT exam, a 1:1 mixture of contrast medium sodium chloride with a flow of 5 ml/s and a delay of 12 s (without thresholding) was chosen. Actual amount of contrast medium was adjusted according to the scan length (100 ml for abdominal aorta only or 120 ml for thoracoabdominal aorta) and the patient weight.

Complications of catheter-directed CTA include local hematoma at the puncture site, dissection of the brachial/axillary/subclavian artery and descending aorta, intravascular thrombosis at the puncture site with potential peripheral thromboembolic complications as well as AV fistula formation. Complications were graded according to the classification of the Society of Interventional Radiology in major and minor [15].

### Image quality

The quality of the acquired CT scans was assessed objectively by comparing signal to noise ratio (from Hounsfield units) in the abdominal aorta at the level of the superior mesenteric artery origin. ROIs were drawn manually within the aorta as well as in the adjacent autochthonous back muscles at the same level. Image noise was defined as standard deviation of the muscle ROI. SNR was calculated as mean signal intensity in the aorta (ROI) divided through the standard deviation of the muscle (ROI).

The subjective image quality was assessed independently by two radiologists with an experience of 4 and 12 years using a 5-point Likert Scale (1 –non-diagnostic, low SNR; 2 –poor quality, limited diagnostic value; 3 –moderate image quality, sufficient SNR for diagnosis; 4 –good image quality, high SNR; 5 –excellent image quality) considering tissue contrast, contrast differences between various tissues as well as pixel level fluctuation and motion artifacts.

### Analysis and statistics

As primary endpoint we compared serum creatinine levels after the CTA (7–30 days), postoperatively after EVAR (7–30 days later) and late after the EVAR (6–60 months). The time gap between the (C) CTA and the laboratory analysis of kidney function (serum creatinine and eGFR) was comparable between the two groups, 7.5 ± 5 for the CTA group and 7.7 ± 6 days for the CCTA group (p = 0.8278).

Secondary endpoints encompassed local complications including puncture site hematoma, artery dissection or occlusion, median nerve lesion and cardio- and cerebrovascular complications including acute coronary symptoms, transient ischemic attack (TIA) defined as cerebral ischemic symptoms with a complete restoration after 24 hours, or stroke defined as cognitive or motoric dysfunction lasting longer than 24h. Statistical analysis was performed using GraphPad Prism version 8 with the appropriate tests (chi square for the assessment of comorbidities, fisher’s exact test for the stages of nephropathy, or unpaired two-tailed t-test for patients’ characteristics, SNR and amount of contrast medium). For the assessment of interobserver variability of image quality the Intraclass Coefficient (ICC) was used, using a two-way random approach ICC(2,k). According to Landis and Koch [16] a ICC/kappa value of k<0.2 is regarded as poor agreement, k = 0.21–0.4 fair agreement, k = 0.41 to 0.6 moderate agreement, k = 0.61 to 0.8 good/substantial agreement, k>0.81 perfect agreement. A p-value <0.05 was considered statistically significant.

## Results

### Patient characteristics

Patient characteristics are presented in Table 1. Of note, cardiovascular comorbidities were not significantly different in the i.v. and catheter-directed group, only hypertension was more common in the catheter-directed group (93 vs. 97%, p = 0.018), which also received more nephrotoxic medication (85 vs. 70%, p = 0.031). Furthermore, the patients in the catheter group had a higher stage of chronic kidney disease (CKD). 34% of patients in the i.v. CTA group that had a normal renal function compared to 0% in the CCTA group (see Table 1, p<0.0001). There were a higher number of patients with a stage 3 and 4 CKD in the CCTA compared to the i.v. CTA group (85% vs. 42%, p<0.0001), indicating that CCTA was predominantly performed in patients with already impaired renal function. The amount of contrast medium needed was 119 ±14 ml in the i.v. CTA group and 24 ± 7 ml in the CCTA group (p<0.0001). For the EVAR procedure, the amount of contrast medium was comparable between the two groups, 122 ± 52 ml for the i.v. CTA group and 109 ± 69 ml for the CCTA group (p = 0.308).

**Table 1:** Patient characteristics.

Characteristics	i.v.	catheter	p value
Number of patients	59	33	
Age Mean	73.6	73.9	0.885^+^
Age IQR	69.5–79.5	71.3–79	
Male	66	31	0.122^+^
Female	4	2	
**Comorbidity**			
Hypertension	55 (93%)	32 (97%)	0.018*
Smoker	36 (61%)	16 (48%)	0.835*
Diabetes	8 (14%)	9 (27%)	0.087*
Hyperlipoproteinaemia	44 (75%)	22 (67%)	1*
Carotis Plaque	6 (10%)	6 (18%)	0.2*
Cardiomyopathy	31 (53%)	21 (64%)	0.143*
CABG	28 (48%)	17 (52%)	0.294*
Nephrotoxic medication	50 (85%)	23 (70%)	0.031*
Nephropathy			
Stage			0.0001*
0	20	0	
I	14	1	
II	12	5	
III	20	16	
IV	5	12	

^+^ t-test, unpaired

*Pearson’s Chi Square Test, 2-sided

Abbreviations: CKD (chronic kidney disease), CABG (coronary artery bypass graft)

### Serum creatinine and eGFR

**Fig 1:**
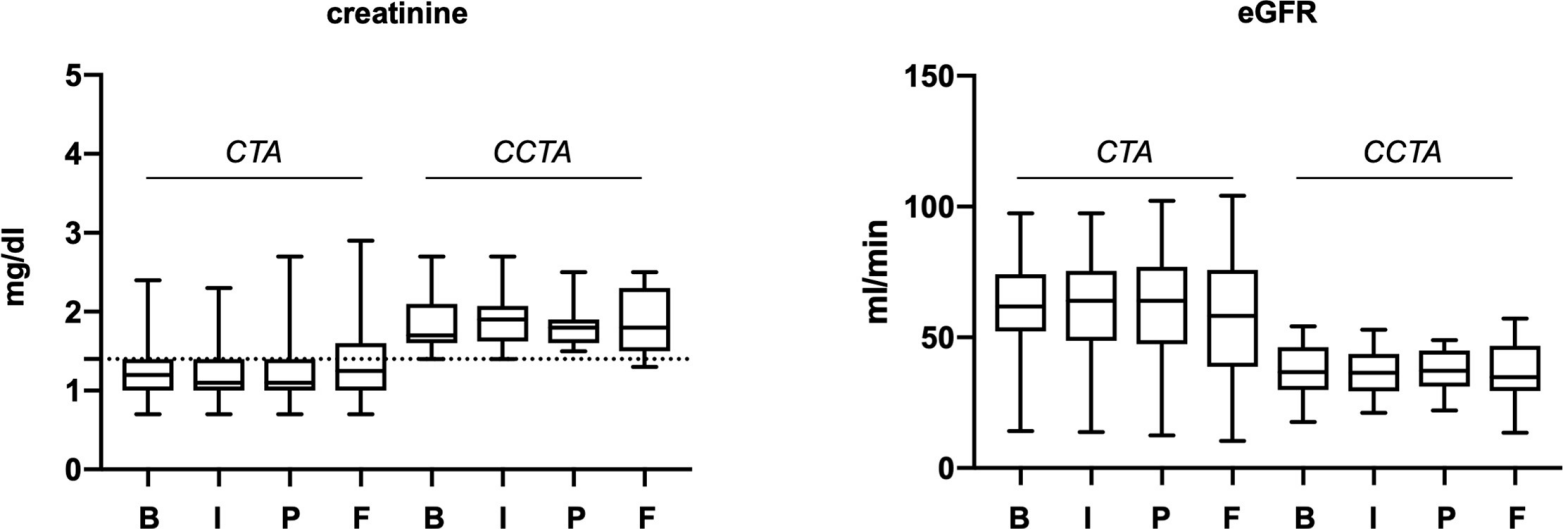
Creatinine levels in the course of CTA at baseline (B), 1–7 days post CT imaging (I), preoperatively before endovascular aortic aneurysm repair (P) and at late follow-up (F). Dotted line marks border towards elevated creatine levels beyond the reference values of 1.4 mg/dl.

As presented above, baseline creatinine levels were higher in the CCTA group, as these patients were considered to have a higher risk of renal function deterioration and were thus more often chosen for the catheter directed CTA. Both groups (i.v. CTA vs. catheter-directed CTA) did not show significant differences in the mean creatinine level after the CT (1.3 ± 0.5 mg/dl, p = 0.912 vs. 1.9 ± 0.4 mg/dl, p = 0.879), preoperatively (1.3 ± 0.6 mg/dl, p = 0.918 vs. 1.9 ± 0.4 mg/dl, p = 0.811) and in the late follow-up (1.4 ± 0.7 mg/dl, p = 0.210 vs. 2.1 ± 0.8 mg/dl, p = 0.387) compared to the baseline values respectively (1.3 ± 0.6 mg/dl vs. 1.9 ± 0.6 mg/dl) (Fig 1, left panel). Comparing eGFR values in the course of EVAR in the same patients similarly revealed no significant differences between the two groups (Fig 1, right panel). Due to the differences in baseline creatinine and eGFR values we performed a matching of 16 patients with similar creatine/eGFR baseline values. This analysis similarly did not reveal differences in the course after EVAR between both groups. Of note, there were single patients in both groups that showed a long-term elevation of serum creatinine ≥0,3 mg/dl compared to their individual baseline level– 15 patients (25%) in the i.v. group vs. two patients in the catheter-directed group (6%). Thus, for individual patients, there may be a potential benefit receiving catheter-directed CTA.

### Image quality

**Fig 2:**
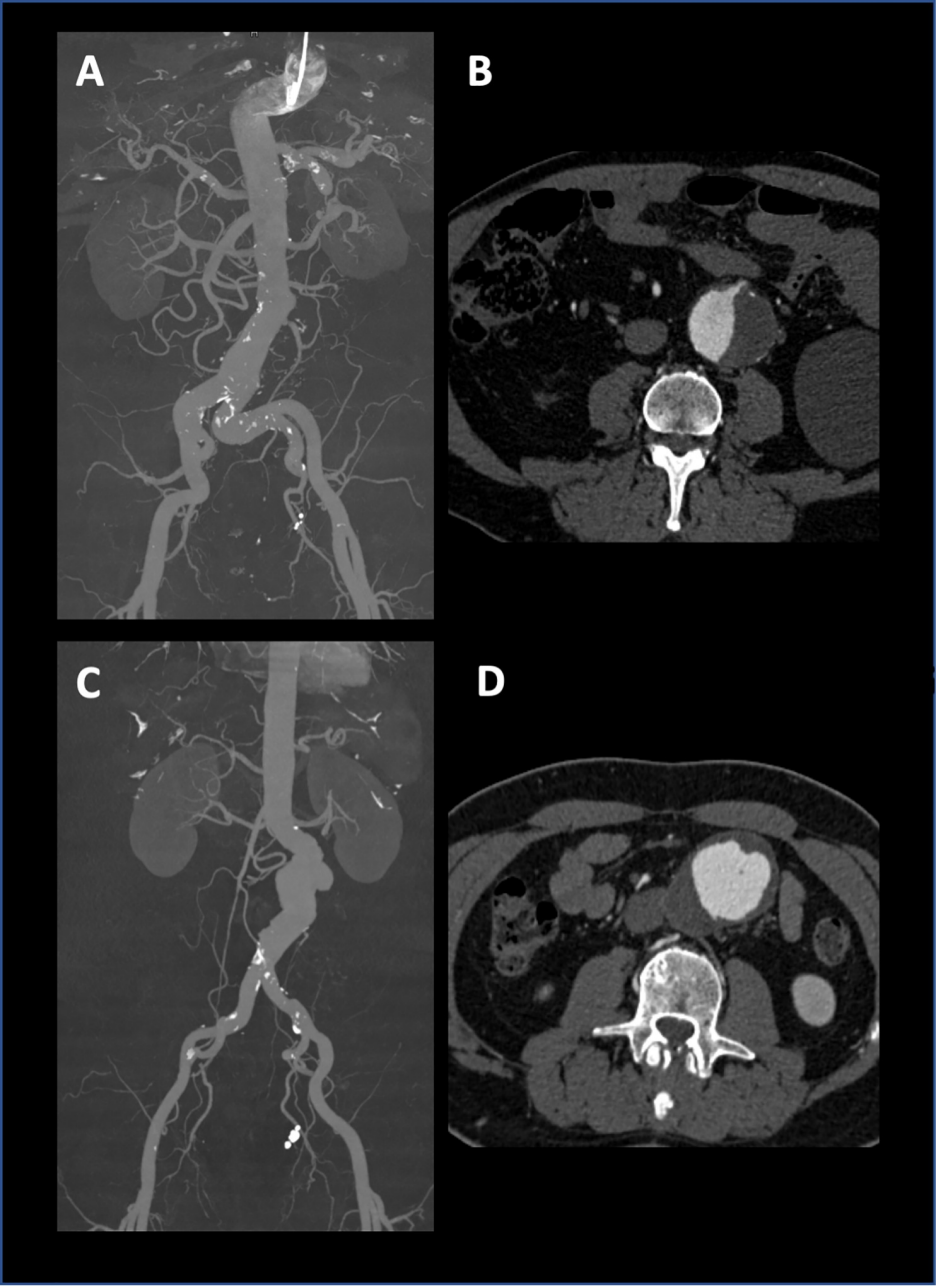
Representative CTA images of a catheter-directed CTA (coronal maximum intensity projection in **A**, transversal image after i.a. contrast application in **B**), and in comparison of an intravenous CTA (coronal maximum intensity projection in **C**, transversal image after i.v. contrast application in **D**).

Signal intensities showed no statistical differences between the two groups (see Table 2). Exemplary images showing a comparable contrast enhancement after i.a. and i.v. contrast media application (Fig 2). Mean image quality rated for the i.v. CTA group was 4.3 ± 0.7 for the first reader, and 3.9 ± 0.7 for reader number 2; for the catheter-directed CTA group 4.2 ± 0.6 for the first reader and 3.9 ± 0.6 for the second reader. The values between the two groups were not significant, with p-values of 0.231 for the first, and 0.816 for the second reader. Interobserver comparison revealed a good agreement with an ICC value of 0.65 [16].

**Table 2:** Comparison of the signal intensities (objective image quality assessment).

	i.v. CTA	Catheter CTA	p-value
SI Aorta	328.1 ± 101	310 ± 136.2	0.471
SI Muscle	42.7 ± 10.8	44 ± 12.1	0.583
Noise	24.3 ± 5.2	25.8 ± 7.0	0.237
SNR	8.5 ± 46	7.7 ± 4.1	0.370

### Complications

There were no major local complications due to intraarterial catheter placement, local hematoma at the puncture site resolved without treatment in n = 7 cases. In our study there was one major stroke in the catheter CTA group which occurred 6 days post catheter CTA periprocedurally due to a thoraco-abdominal aortic dissection during EVAR, which was therefore not related to placement of the Pigtail catheter for CTA.

All raw data are provided in S1 File.

## Discussion

For the past years, the use of iodine-based contrast medium has been described as a risk factor for renal function deterioration and a large number of studies focusing on treating and preventing the incidence of this complication were published [17–20]. Several techniques for the prophylaxis of CIN after the use of contrast medium have been reported, including the use of saline hydration with or without acetyl-cysteine, ischemia-reperfusion reconditioning, or the use of high dose statins. A recent study analyzed patients at high risk for renal complications who were undergoing angiography, regarding prevention of death, need for dialysis, persistent decline in kidney function at 90 days and prevention of contrast-associated acute kidney injury (PRESERVE Trial). This study found no benefit of intravenous sodium bicarbonate over intravenous sodium chloride or of oral acetylcysteine over placebo [21].

Until now, the mechanisms of CIN after application of contrast medium still remains a feared but not fully understood complication, which is associated with poor in-hospital outcomes [22–25]. Therefore, we introduced a contrast sparing intraarterial CT-angiography for patients with reduced renal function to avoid impairment of kidney function at our institution in 2009. Comparing catheter directed CTA to CTA with i.v. contrast media application revealed that both achieve a comparable, fully diagnostic image quality with a significantly reduced amount of contrast medium required for CCTA. However, this reduced amount of contrast media was obviously not associated with altered renal function. This absent protective effect on renal function has also been observed by Isaacson et al., Garcarek et al. and Swanberg et al. [12, 13, 26].

Similar results were reported in a study proposing this technique for octogenarians being evaluated for transarterial aortic valve replacement. However, in their cohort there was no significant difference in rates of CIN after CTA between standard and low-dose contrast protocol groups, with no CIN events in those imaged by low-dose CTA [27]. However, according to the contrast-induced nephropathy consensus working panel, the intraarterial delivery of iodinated contrast agents poses a higher risk for CIN [28].

Most published data refer to CIN following cardiac arteriography examinations exposing the kidneys not only to repeated concentrated contrast medium boli but also potential atheroemboli. Interestingly, Gohbara et al. recently demonstrated, that the incidence of acidosis in patients with STEMI and reperfusion within 12 hours of symptom onset is associated with a higher incidence of CIN [29]. Other authors postulated the hypothesis that an acute congestive heart failure or cardiogenic shock may cause renal dysfunction attributable to acute kidney injury (AKI) [30]. Therefore, contrast medium exposure alone does not explain the elevated risk for an acute kidney injury after coronary angiography. In our population, there was no significant difference in incidence of chronic congestive heart failure or previous history of coronary artery bypass between the two groups.

Lately published literature from larger controlled studies suggests no increased risk of CIN after intravenous CM application for routine imaging in patients with normal renal function, but there may be a risk in patients with preexisting kidney injury [31]. A meta-analysis on controlled contrast medium–induced CKD studies demonstrates a similar incidence of acute kidney injury, dialysis, and death between the contrast medium group and control groups [32].

Besides good outcome in our study in terms of lack of complications associated with arterial catheter placement, there are also disadvantages using this technique. There are higher expenses associated with this technique, with the need for a sterile intervention unit and experienced interventionalists.

Limitations include the retrospective character of this study, performed at a single-center only. The fact that patients with a catheter-directed CTA generally had a higher serum creatinine baseline level and elevated stages of nephropathy, makes the two groups less comparable.

## Conclusion

Catheter-directed intraarterial contrast medium application is safe and offers the possibility to obtain CT angiographies with a low amount of contrast medium but achieving a comparable image quality compart to standard CTA. However, as there is no proven benefit regarding renal function preservation and higher expenses for a catheter-directed CTA, the procedure cannot be generally recommended for clinical routine and should only be restricted to patients who are at high risk for renal function deterioration or have shown such deterioration following contrast media application before. Further research needs to be initiated to identify individual patients who might benefit from this approach.

## Supporting information

S1 File Supplement. (XLSX)

## References

[1] Ashton HA, Buxton MJ, Day NE, Kim LG, Marteau TM, Scott RAP, Thompson SG, Walker NM (2002) The Multicentre Aneurysm Screening Study (MASS) into the effect of abdominal aortic aneurysm screening on mortality in men: a randomised controlled trial. Lancet 360(9345):1531–1539 10.1016/s0140-6736(02)11522-4 12443589

[2] Gianfagna F, Veronesi G, Bertù L, Tozzi M, Tarallo A, Ferrario MM, Castelli P (2016) Prevalence of abdominal aortic aneurysms and its relation with cardiovascular risk stratification: protocol of the Risk of Cardiovascular diseases and abdominal aortic Aneurysm in Varese (RoCAV) population based study. BMC Cardiovasc Disord 16(1):243 10.1186/s12872-016-0420-2 27894269 PMC5127056

[3] Kniemeyer HW, Kessler T, Reber PU, Ris HB, Hakki H, Widmer MK (2000) Treatment of ruptured abdominal aortic aneurysm, a permanent challenge or a waste of resources? Prediction of outcome using a multi-organ-dysfunction score. Eur J Vasc Endovasc Surg 19(2):190–196. 10.1053/ejvs.1999.0980 10727370

[4] Wanhainen A, Verzini F, van Herzeele I et al (2019) Editor's Choice—European Society for Vascular Surgery (ESVS) 2019 Clinical Practice Guidelines on the Management of Abdominal Aorto-iliac Artery Aneurysms. Eur J Vasc Endovasc Surg 57(1):8–93. 10.1016/j.ejvs.2018.09.020 30528142

[5] Anderson PL, Arons RR, Moskowitz AJ, Gelijns A, Magnell C, Faries PL, Clair D, Nowygrod R, Kent KC (2004) A statewide experience with endovascular abdominal aortic aneurysm repair: rapid diffusion with excellent early results. J Vasc Surg 39(1):10–19. 10.1016/j.jvs.2003.07.020 14718804

[6] Powell JT, Sweeting MJ, Ulug P, Blankensteijn JD, Lederle FA, Becquemin J-P, Greenhalgh RM (2017) Meta-analysis of individual-patient data from EVAR-1, DREAM, OVER and ACE trials comparing outcomes of endovascular or open repair for abdominal aortic aneurysm over 5 years. Br J Surg 104(3):166–178. 10.1002/bjs.10430 28160528 PMC5299468

[7] Zettervall SL, Ultee KHJ, Soden PA, Deery SE, Shean KE, Pothof AB, Wyers M, Schermerhorn ML (2017) Predictors of renal dysfunction after endovascular and open repair of abdominal aortic aneurysms. J Vasc Surg 65(4):991–996. 10.1016/j.jvs.2016.06.113 27687321 PMC5366267

[8] Dang T, Dakour-Aridi H, Rizwan M, Nejim B, Malas MB (2019) Predictors of acute kidney injury after infrarenal abdominal aortic aneurysm repair in octogenarians. J Vasc Surg 69(3):752–762.e1. 10.1016/j.jvs.2018.05.227 30154014

[9] Adas Z al Shepard AD, Nypaver TJ Weaver MR, Maatman T Yessayan LT, Balraj P Kabbani LS (2018) Long-term decline in renal function is more significant after endovascular repair of infrarenal abdominal aortic aneurysms. J Vasc Surg 68(3):739–748. 10.1016/j.jvs.2017.12.051 29571627

[10] Chaikof EL, Dalman RL, Eskandari MK, Jackson BM, Lee WA, Mansour MA, Mastracci TM, Mell M, Murad MH, Nguyen LL, Oderich GS, Patel MS, Schermerhorn ML, Starnes BW (2018) The Society for Vascular Surgery practice guidelines on the care of patients with an abdominal aortic aneurysm. J Vasc Surg 67(1):2–77.e2. 10.1016/j.jvs.2017.10.044 29268916

[11] Faggioni M, Mehran R (2016) Preventing Contrast-induced Renal Failure: A Guide. Interventional Cardiology Review 11(2):98 10.15420/icr.2016:10:2 29588714 PMC5808627

[12] Isaacson AJ, Burke LMB, Vallabhaneni R, Farber MA (2016) Ultralow Iodine Dose Transarterial Catheter-Directed CT Angiography for Fenestrated Endovascular Aortic Repair Planning. Ann Vasc Surg 35:234–237. 10.1016/j.avsg.2016.01.045 27238979

[13] Garcarek J, Kurcz J, Guziński M, Banasik M, Miś M, Gołębiowski T (2015) Intraarterial CT Angiography Using Ultra Low Volume of Iodine Contrast—Own Experiences. Pol J Radiol 80:344–349. 10.12659/PJR.894050 26191113 PMC4497469

[14] Formosa A, Santos DM, Marcuzzi D, Common AA, Prabhudesai V (2016) Low Contrast Dose Catheter-Directed CT Angiography (CCTA). Cardiovasc Intervent Radiol 39(4):606–610. 10.1007/s00270-015-1232-y 26514834

[15] Khalilzadeh O, Baerlocher MO, Shyn PB, Connolly BL, Devane AM, Morris CS, Cohen AM, Midia M, Thornton RH, Gross K, Caplin DM, Aeron G, Misra S, Patel NH, Walker TG, Martinez-Salazar G, Silberzweig JE, Nikolic B (2017) Proposal of a New Adverse Event Classification by the Society of Interventional Radiology Standards of Practice Committee. J Vasc Interv Radiol 28(10):1432–1437.e3. 10.1016/j.jvir.2017.06.019 28757285

[16] Landis JR, Koch GG (1977) The measurement of observer agreement for categorical data. Biometrics 33(1):159–174 843571

[17] Zhang B, Liang L, Chen W, Liang C, Zhang S (2015) The efficacy of sodium bicarbonate in preventing contrast-induced nephropathy in patients with pre-existing renal insufficiency: a meta-analysis. BMJ Open 5(3):e006989 10.1136/bmjopen-2014-006989 25783425 PMC4368906

[18] Subramaniam RM, Suarez-Cuervo C, Wilson RF, Turban S, Zhang A, Sherrod C, Aboagye J, Eng J, Choi MJ, Hutfless S, Bass EB (2016) Effectiveness of Prevention Strategies for Contrast-Induced Nephropathy: A Systematic Review and Meta-analysis. Ann Intern Med 164(6):406–416. 10.7326/M15-1456 26830221

[19] Xu R, Tao A, Bai Y, Deng Y, Chen G (2016) Effectiveness of N-Acetylcysteine for the Prevention of Contrast-Induced Nephropathy: A Systematic Review and Meta-Analysis of Randomized Controlled Trials. J Am Heart Assoc 5(9). 10.1161/JAHA.116.003968 27663415 PMC5079043

[20] Weisbord SD, Gallagher M, Kaufman J, Cass A, Parikh CR, Chertow GM, Shunk KA, McCullough PA, Fine MJ, Mor MK, Lew RA, Huang GD, Conner TA, Brophy MT, Lee J, Soliva S, Palevsky PM (2013) Prevention of contrast-induced AKI: a review of published trials and the design of the prevention of serious adverse events following angiography (PRESERVE) trial. Clin J Am Soc Nephrol 8(9):1618–1631. 10.2215/CJN.11161012 23660180 PMC3805082

[21] Weisbord SD, Gallagher M, Jneid H, Garcia S, Cass A, Thwin S-S, Conner TA, Chertow GM, Bhatt DL, Shunk K, Parikh CR, McFalls EO, Brophy M, Ferguson R, Wu H, Androsenko M, Myles J, Kaufman J, Palevsky PM (2018) Outcomes after Angiography with Sodium Bicarbonate and Acetylcysteine. N Engl J Med 378(7):603–614. 10.1056/NEJMoa1710933 29130810

[22] Nakahashi H, Kosuge M, Sakamaki K, Kiyokuni M, Ebina T, Hibi K, Tsukahara K, Iwahashi N, Kuji S, Oba MS, Umemura S, Kimura K (2017) Combined impact of chronic kidney disease and contrast-induced nephropathy on long-term outcomes in patients with ST-segment elevation acute myocardial infarction who undergo primary percutaneous coronary intervention. Heart Vessels 32(1):22–29. 10.1007/s00380-016-0836-8 27106917

[23] Giacoppo D, Madhavan MV, Baber U, Warren J, Bansilal S, Witzenbichler B, Dangas GD, Kirtane AJ, Xu K, Kornowski R, Brener SJ, Généreux P, Stone GW, Mehran R (2015) Impact of Contrast-Induced Acute Kidney Injury After Percutaneous Coronary Intervention on Short- and Long-Term Outcomes: Pooled Analysis From the HORIZONS-AMI and ACUITY Trials. Circ Cardiovasc Interv 8(8):e002475 10.1161/CIRCINTERVENTIONS.114.002475 26198286

[24] Watabe H, Sato A, Hoshi T, Takeyasu N, Abe D, Akiyama D, Kakefuda Y, Nishina H, Noguchi Y, Aonuma K (2014) Association of contrast-induced acute kidney injury with long-term cardiovascular events in acute coronary syndrome patients with chronic kidney disease undergoing emergent percutaneous coronary intervention. Int J Cardiol 174(1):57–63. 10.1016/j.ijcard.2014.03.146 24726211

[25] Narula A, Mehran R, Weisz G, Dangas GD, Yu J, Généreux P, Nikolsky E, Brener SJ, Witzenbichler B, Guagliumi G, Clark AE, Fahy M, Xu K, Brodie BR, Stone GW (2014) Contrast-induced acute kidney injury after primary percutaneous coronary intervention: results from the HORIZONS-AMI substudy. Eur Heart J 35(23):1533–1540. 10.1093/eurheartj/ehu063 24603308

[26] Swanberg J, Nyman R, Magnusson A, Wanhainen A (2014) Selective intra-arterial dual-energy CT angiography (s-CTA) in lower extremity arterial occlusive disease. Eur J Vasc Endovasc Surg 48(3):325–329. 10.1016/j.ejvs.2014.05.013 24958221

[27] Zemedkun M, LaBounty TM, Bergman G, Wong S-C, Lin FY, Reynolds D, Gomez M, Dunning AM, Leipsic J, Min JK (2015) Effectiveness of a low contrast load CT angiography protocol in octogenarians and nonagenarians being evaluated for transcatheter aortic valve replacement. Clin Imaging 39(5):815–819. 10.1016/j.clinimag.2014.08.010 25982494

[28] McCullough PA (2008) Contrast-induced acute kidney injury. J Am Coll Cardiol 51(15):1419–1428. 10.1016/j.jacc.2007.12.035 18402894

[29] Gohbara M, Hayakawa A, Akazawa Y, Furihata S, Kondo A, Fukushima Y, Tomari S, Endo T, Kimura K, Tamura K (2017) Association Between Acidosis Soon After Reperfusion and Contrast-Induced Nephropathy in Patients With a First-Time ST-Segment Elevation Myocardial Infarction. J Am Heart Assoc 6(8). 10.1161/JAHA.117.006380 28835362 PMC5586466

[30] Andò G, Morabito G, Gregorio C de, Trio O, Saporito F, Oreto G (2013) Age, glomerular filtration rate, ejection fraction, and the AGEF score predict contrast-induced nephropathy in patients with acute myocardial infarction undergoing primary percutaneous coronary intervention. Catheter Cardiovasc Interv 82(6):878–885. 10.1002/ccd.25023 23703775

[31] McDonald JS, McDonald RJ, Comin J, Williamson EE, Katzberg RW, Murad MH, Kallmes DF (2013) Frequency of acute kidney injury following intravenous contrast medium administration: a systematic review and meta-analysis. Radiology 267(1):119–128. 10.1148/radiol.12121460 23319662

[32] Ghumman SS, Weinerman J, Khan A, Cheema MS, Garcia M, Levin D, Suri R, Prasad A (2017) Contrast induced-acute kidney injury following peripheral angiography with carbon dioxide versus iodinated contrast media: A meta-analysis and systematic review of current literature. Catheter Cardiovasc Interv 90(3):437–448. 10.1002/ccd.27051 28463460

